# Brain Structural Bases of Tendency to Forgive: evidence from a young adults sample using voxel-based morphometry

**DOI:** 10.1038/s41598-017-16868-3

**Published:** 2017-12-04

**Authors:** Haijiang Li, Qunlin Chen, Jiamei Lu, Jiang Qiu

**Affiliations:** 1 Department of Psychology, Shanghai Normal University, Shanghai, 200234 China; 2 Key Laboratory of Cognition and Personality (SWU), Ministry of Education, Chongqing, 400715 China; 3 Faculty of Psychology, Southwest University, Chongqing, 400715 China

## Abstract

Tendency to forgive refers to one’s global dispositional level of forgiveness across situations and relationships. Brain imaging studies examined activation patterns underlying forgiving response, yet focal differences in brain structures related to tendency to forgive have never been investigated. In this study, voxel-based morphometry was used to investigate relations between gray matter/white matter volume (GMV/WMV) and individual differences in tendency to forgive in a large young sample. Participants were 199 young students (60 men) who completed the tendency to forgive scale (TTF) and underwent an anatomical magnetic resonance imaging scan. Results showed that higher TTF scores were associated with larger GMV in the regions of dorsolateral prefrontal cortex, and smaller GMV in the regions of the right insular cortex and inferior frontal gyrus (IFG). Moreover, higher TTF scores were also related to smaller WMV in the regions of the left IFG. Together, these findings suggest structural variations for individual differences in the tendency to forgive, distributed across different brain regions associated with empathic response and cognitive control.

## Introduction

In recent decades, scientific investigations of forgiveness have a rapid increase from many perspectives within psychology^1,2^. Forgiveness is a complex phenomenon which reflects by there is a lack of consensus on exact forgiveness definition^3–5^. However, most researchers would agree that forgiveness involved prosocial changes toward the transgressor, including cognitive, affective, behavioral or motivational changes^1,6^. The tendency to forgive refers to one’s global dispositional level of forgiveness across situations and relationships^7^. Previous studies have linked low levels of tendency to forgive not only to mental disturbances like neuroticism^8^, depression^7^ and rumination^9^, but also to physical health outcomes including increased sleep issues, hypertension, and risk of cardiovascular disease^10–12^.

Task-based functional magnetic resonance imaging (fMRI) studies exploring neural response to transgression have found activation of the lateral prefrontal cortex (PFC), medial PFC, anterior cingulate cortex (ACC), temporoparietal junction (TPJ), and insular cortex which played an important role in cognitive control, empathic response, and negative affect states^13–17^. Farrow *et al*. first reported that forgiving judgments of one’s crime elicited increased activation of superior frontal gyrus^13^ and posterior cingulate cortex. Using similar paradigm, researchers found that granting forgiveness was related to increased activation of dorsolateral PFC (DLPFC), inferior parietal lobule, and precuneus which were implicated in cognitive control and empathy^15^.

Except for forgiving judgment and imagination, researchers also explored brain activation patterns associated with forgiving response using behavioral measures of forgiveness. For instance, researchers investigated the neural correlates of forgiveness and unforgiveness (i.e. punishment) of initiators of social exclusion and found that refraining from punishment (i.e. forgiveness) was related to increased activation in brain regions concerning theory of mind, such as TPJ, dorsomedial PFC, and inferior frontal gyrus (IFG), and in brain regions involved cognitive control including dorsal ACC, and DLPFC^16^. Consistent with this, researchers also found that granting forgiveness for participants who previously excluded them was related to activation of DLPFC in normal individuals^18^ and individuals experienced chronically rejected and stably highly accepted^17^, suggesting cognitive control and emotional regulation of DLPFC to the anger and resentment to transgressor is important in granting forgiveness.

Additionally, inferring and understanding the intention and behavior of perpetrators is a key process of forgiveness, a recent study on intention understanding emphasized the role of the insular cortex and IFG in the emotional responses to transgression and subsequent forgiveness^19^. Higher trait forgiveness evidenced lower anterior insular activation when finding out an innocent intention after being accidentally harmed^19^. While unforgiving response (i.e. punishment) were correlated with increased activation in the anterior insular^16,20^, reflecting an important role of the insular cortex in processing aversive responses to interpersonal transgressions^21^. A recent study also attempted to investigate neuroanatomical associations of forgiving within a brain network involved in metalizing which was regarded as a key variable in the process of forgiveness^5,22^. Results found a positive correlation between grey matter volume in the superior temporal sulcus and forgiving response to accidental harms^23^.

In large part, previous research mainly examined activation patterns underlying the granting forgiveness. However, focal differences in brain structures, i.e., gray matter volume (GMV) and white matter volume (WMV) related to individual differences in tendency to forgive, have never been directly investigated. Research on links between GMV/WMV and individual difference in tendency to forgive is important in helping to deepen our understanding of the neural substrates contributing to the willingness to forgive. Thus, the aim of the present study is to explore direct associations between individual differences in tendency to forgive measured by tendency to forgive scale and neuroanatomical differences in GMV and WMV using voxel-based morphometry (VBM). VBM is an objective, reliable method that eliminates effects of operator bias^24^. Given its task-free conditions, VBM analysis has been widely used to examine the brain substrate of the psychological traits, such as intelligence, personality and emotional states^25–27^. Drawing upon findings from prior imaging studies, we posited that individual variations in tendency to forgive would be associated GMV/WMV differences in brain regions implicated in cognitive control, empathy and aversive responses, such as lateral PFC, ACC, medial PFC, TPJ, and insular cortex.

## Results

### Tendency to Forgive (TTF) Scores

The mean TFF scores for the current sample was 13.37 (SD = 2.30) with a range of 7 to 19. No significant gender difference in TTF scores was found in the present study [*t* (197) = 1.33, *p* = 0.19].

### Associations between GMV and TTF scores

**Figure 1:**
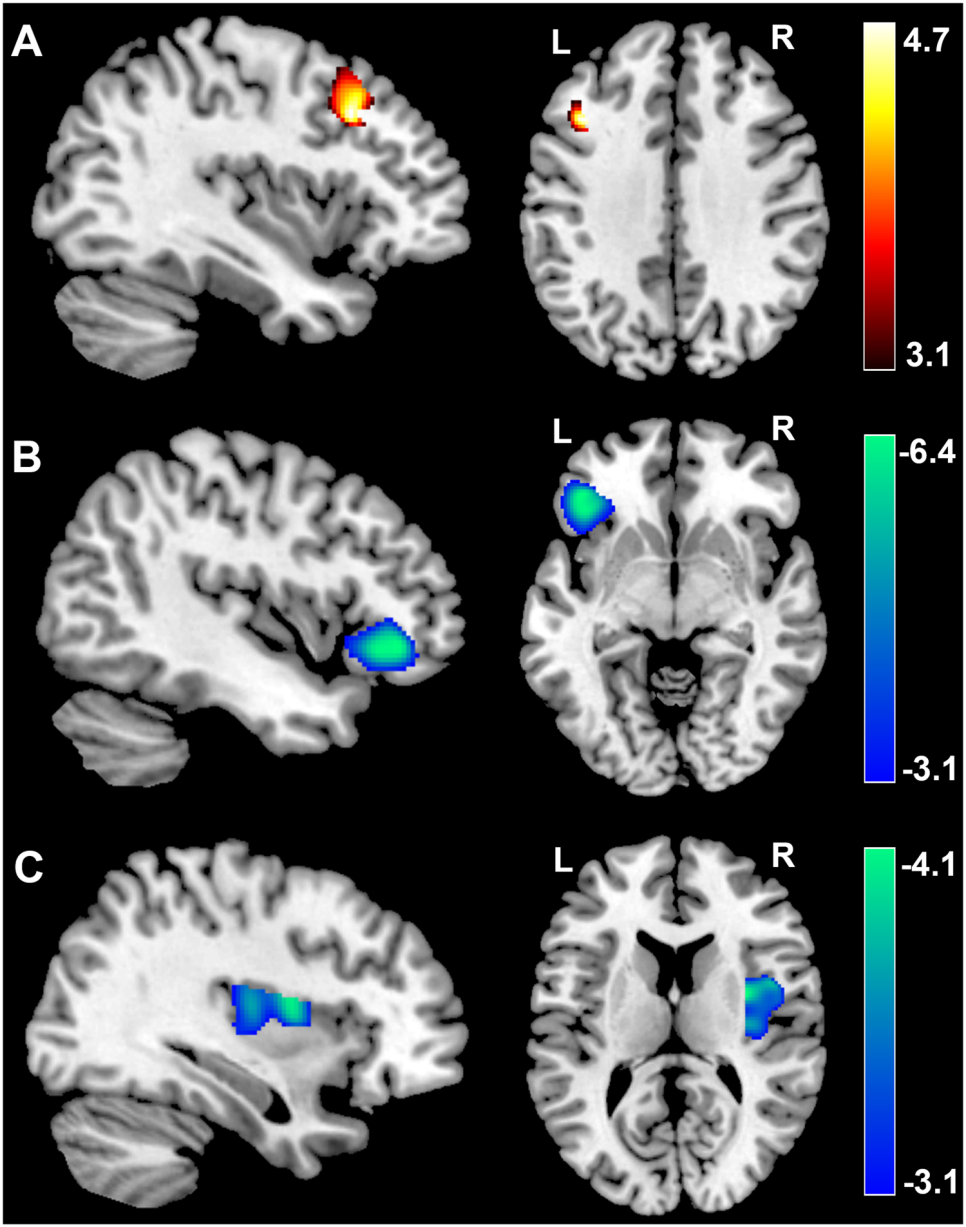
Association between TTF scores and GMV. GMV of the dorsolateral prefrontal cortex were positively correlated with TTF scores (**A**). GMV in the left IFG had a significant, negative correlation with TTF scores (**B**). GMV in the right insular cortex had a significant, negative correlation with TTF scores (**C**). Results are shown at p < 0.05, corrected for multiple comparisons at the cluster-level with non-stationary correction and an underlying voxel level of P < 0.001, uncorrected. The color density represents the T score. TTF: tendency to forgive; GMV: gray matter volume; IFG, inferior frontal gyrus; L, left; R, right.

After correcting for age, sex and total brain gray matter volume, TTF scores had significant, positive associations with GMV in a cluster that included regions of the left dorsolateral prefrontal cortex [MNI coordinate: −39, 20, 36; *t* = 4.64; *p* (corrected) < 0.05; Cluster size = 1812, Table 1; Fig. 1A]. Furthermore, TTF scores had a significant, negative correlation with GMV in a cluster that mainly included the left inferior frontal cortex [MNI coordinate: −44, 36, −6; *t* = 6.38; Cluster size = 4941; *p* (corrected) < 0.05, Table 1; Fig. 1B] and in a cluster that mainly included the right insular cortex [MNI coordinate: 33, 0, 12; *t* = 4.07; *p* (corrected) < 0.05; Cluster size = 4847, Table 1; Fig. 1C]. No other significant relations were observed.

**Table 1:** Brain regions with significant association between brain structures and TTF scores.

Brain regions	BA	MNI coordination			Cluster size (mm^3^)	Peak T-Value
		x	y	z		
Correlation between TTF and GMV						
*Positive correlations*						
DLPFC	9	−39	20	36	1812	4.64
*Negative correlation*						
IFG	47	−44	36	−6	4941	6.38
Insular cortex	13	33	0	12	4847	4.07
Correlation between TTF and WMV						
*Negative correlation*						
IFG		−42	39	3	2585	4.86

TTF, tendency to forgive; DLPFC, dorsolateral prefrontal cortex; IFG, inferior frontal gyrus; GMV, gray matter volume; WMV, white matter volume; BA, broadman area.

### Associations between WMV and TTF scores

**Figure 2:**
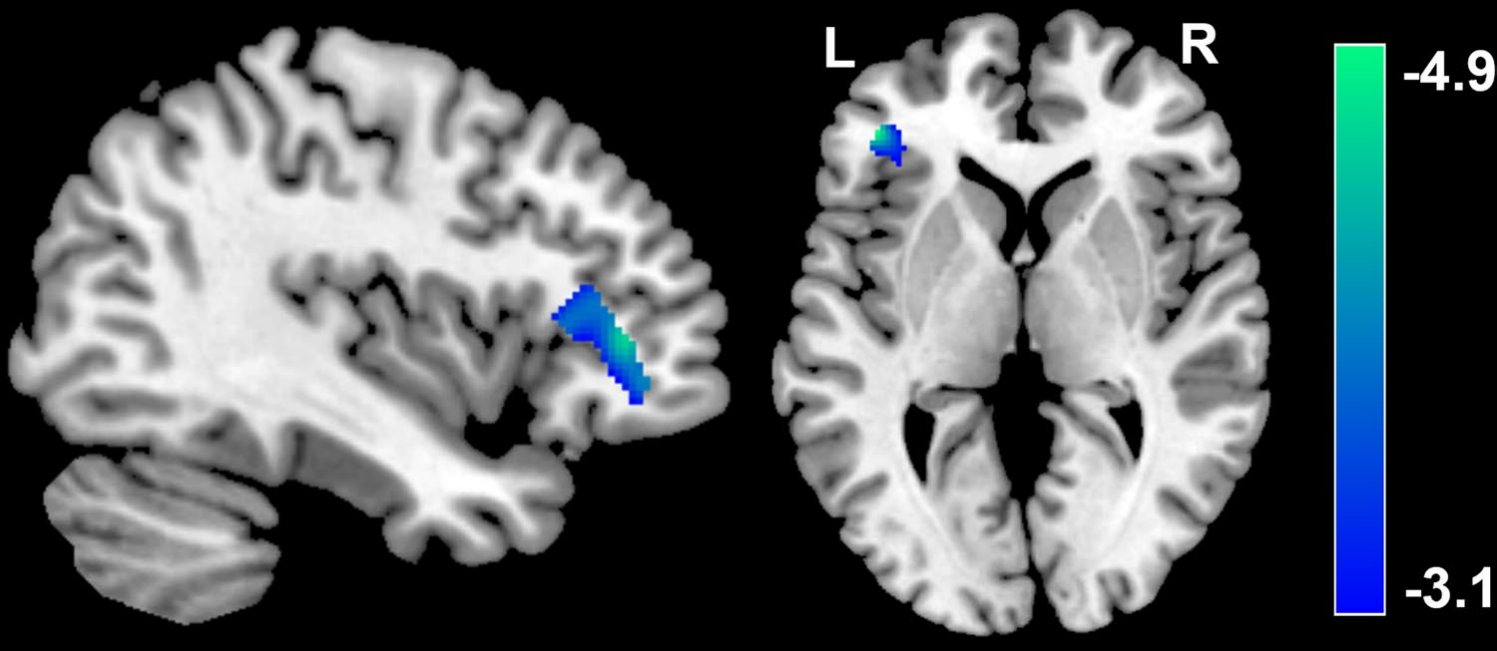
Association between TTF scores and WMV. The left IFG had a significant and negative correlation with TTF scores. Results are shown at p < 0.05, corrected for multiple comparisons at the cluster-level, with an underlying voxel level of P < 0.001, uncorrected. The color density represents the T score. TTF: tendency to forgive; WMV: white matter volume; IFG, inferior frontal gyrus; L, left; R, right.

After correcting for age, sex and total brain white matter volume, TTF scores had a significant, negative correlation with WMV in a cluster that included the left inferior frontal cortex [MNI coordinate: −42, 39, 3; *t* = 4.86; Cluster size = 2585; *p* (corrected) < 0.05, Table 1; Fig. 2].No other significant associations were found.

## Discussion

In the current study, we explored the neuroanatomical associations of individual differences of tendency to forgive using voxel-based morphometry in a large young sample. Consistent with the general hypothesis that the TTF scores would vary as a function of GMV and WMV, tendency to forgive was (a) positively associated with GMV in a cluster that mainly included the left dorsolateral prefrontal cortex (DLPFC); (b) negatively associated with GMV in a cluster that mainly including the right inferior frontal gyrus (IFG) and in the region of right insular cortex, and negatively correlated with WMV in a cluster that included the right IFG. These results provided direct neuroanatomical evidence for the association between tendency to forgive and brain regions that are important for cognitive control, empathic processing, and aversive responses.

Increased GMV in a cluster included the DLPFC was observed in participants with higher TTF scores. The DLPFC was suggested to play an important role in the cognitive control^28^, moral decision making^29^, and emotional regulation^30^. Dysfunction to DLPFC has been linked to major depression disorder^31^, substance abuse^32^ and post-traumatic stress disorder^33^. Imaging studies found that when participants expressed forgiving behavior to people who previously transgressed them would elicit increased activation of the DLPFC^15,16,18^, suggesting its effect on conflict-induced behavioral adjustment. This was also supported by study found that when individuals confronted those who have previously exhibited non cooperative behavior showed greater activation of the DLPFC^34^. Additionally, forgiveness therapy assumed that helping people overcome unforgiveness (i.e. resentment, bitterness) toward those who have transgressed them is one primary process of granting forgiveness^22^. And increased activity in the DLPFC leads to a greater regulatory role in attentional deployment toward threatening stimuli^35^. Thus, these studies and current findings suggested that increased GMV in regions of the DLFPC may facilitate participants with higher TTF scores controlling and regulating their prepotent responses (i.e. retaliation, bitterness) against transgression resulting in more willing to grant forgiveness.

Individual differences of tendency to forgive were negatively associated with GMV in the regions of right insular cortex and left inferior frontal gyrus (IFG), suggesting participants with increased GMV in the regions of right insula and left IFG are more unforgiving for one’s transgression. The insula is located at the interface of cognitive, homeostatic, and affective systems of the human brain 36 (Menon & Uddin, 2010), and has been involved in a wide range of functions including interoceptive processing, emotional response, and empathic processing^25,37–39^. Previous studies frequently reported the activation of insula cortex in the processing of different aversive events including pain, disgust, and unfariness^40^. When received unfair offers relative to fair offers, increased activation of anterior insula were reported in participants who acted as recipients in an Ultimatum Game^41^. Unforgiving responses (i.e. punishment) to participants who previously excluded them during a virtual ball-tossing game was related to increased activation in the insula cortex^16^, pointing to an important role for the insular cortex in processing aversive responses to moral or interpersonal transgressions^14,18,19,21^. Results from justice sensitivity, which is regarded as a dispositional variable that reflects ones’ concern for justice and is an important predictor of justice-related emotion and behavior^42^, found that individuals with high levels of justice sensitivity for the other rated the immoral behaviors as less permissible during a moral judgment task^43^. Moreover, structural imaging studies on justice sensitivity discovered a negative relation between gray matter volume of insular cortex and justice sensitivity, suggesting the greater of gray matter volume of the insula, the higher sensitivity to injustice experienced by others^44^. The negative association between GMV of the insular cortex and tendency to forgive consistent with the notion that insula cortex plays an important role in the coding of aversive value and injustice sensitivity, suggesting individuals with increased GMV of the insular cortex may have an elevated sensitivity toward injustice which leads individuals to tend to choose unforgiveness in response to interpersonal transgressions.

The present study also observed a negative association between inferior frontal gyrus (IFG) GMV with participants who reported higher TTF scores. The IFG is considered to be involved in inhibitory control^45^ and empathy-related processing, especially emotional aspects of empathy^46^. A weakness of IFG will affect many types of inhibitory response control including attentional control in neuropsychiatric disorders^45^. Forgive-related study found that forgiving responses was correlated to individual differences in empathy and increased activation in the regions of IFG and other regions, suggesting a vital role of IFG in the process of empathy in responding to transgressors^16^. Consistent with this, the prior study observed that receiving an apology from the previous offender was considered will trigger concern for the offender and evoked increased activation of IFG and promoted more forgiveness granting^47^. In addition, brain lesions study revealed that participants with lesions in the IFG showed significant deficits in emotional empathy^48^. Structural imaging study explored structural bases of individual differences in empathic abilities and found that greater empathic abilities towards other person were associated with lower GMV in IFG^49^. Thus, the decreased GMV in the region of IFG in individuals with higher TTF scores may suggest an increased empathic function of IFG which may facilitate increased tendency to forgive.

Decreased regional WMV in the regions of IFG in participants with higher TTF scores may reflect abnormal fibers connections that influence neural transmission in the area and among networks. The IFG is a core region of mirror neuron system, a neural network that is engaged in understanding the actions of other people, learning new skills by imitation, and contributing to empathic abilities^47,50–52^. Consistent with this, diffusion-tensor imaging study found that disrupted white matter integrity of IFG constitutes a pathology underpinning of empathic disabilities in schizophrenia^53^. Additionally, research discovered a significant relationship between emotional empathy and whiter matter microstructure in the inferior fronto-occipital fasciculus^54^, which connects the ventral occipital cortex with the IFG and fronto-orbital cortices^55^. Thus, the enhanced WMV in IFG may affect the functional interactions between IFG and other related regions which may underlie correlations between emotional empathy and tendency to forgive.

In addition, cortical thickness analysis was also conducted to complement the VBM findings. The results observed associations between TTF scale and cortical thickness in the regions that the VBM analysis shows. Individual differences of TTF were positively associated with the cortical thickness of DLPFC and were negatively related to the cortical thickness of IFG and insular cortex (see Supplementary Table 1). However, the results of cortical thickness analysis did not achieve statistical significance. Thus, caution is warranted in interpreting the cortical thickness results.

Notwithstanding its potential implications, the main limitations of this study should be mentioned. First, in light of the cross-sectional research design, causal relations could not be drawn between tendency to forgive and brain structures. Hence, it is not clear whether GMV/WMV differences caused or resulted from high levels of tendency to forgiveness or whether causal associations are reciprocal. Longitudinal designs may be useful in assessing the status of GMV and WMV as predisposing factors that increase susceptibility for later increases in granting forgiveness. Second, the findings that reduced brain volume in the IFG and insular cortex were related to increased levels of tendency to forgive may seem paradoxical to the notion that more-is-better. While such smaller volume with better functions is not uncommon^56,57^ and may reflect developmental cortical thinning after young adolescence^56,58^. Thus, it is conceivable that decreased brain volume as a result of the cortical pruning leads to matured cognitive abilities. Third, caution is warranted in interpreting the results concerning the high level phenomenon like forgiveness. We only assessed individual differences in tendency to forgive which reflects one’s global dispositional level of forgiveness across situations and relationships. Whether the results consistent with the process of granting forgiveness prompt more studies to explore the associations between brain structure and forgiving process. Four, although findings were striking in that variability in GMV and WMV was linked to TTF scores in a young adult cohort drawn from a non-clinical setting, it is not clear whether findings generalize to other age groups or general populations in which TTF scores, GMV and WMV, are more normally-distributed. Finally, findings should be considered provisional. While other GMV and WMV differences corresponding to varying of willing to forgive were not revealed or did not survive corrections for multiple comparisons, replications in other samples are needed to demonstrate the reliability of findings across groups.

In conclusion, this appears to be the first study to directly identify associations between GMV and WMV in particular regions and elevations in tendency to forgive. Specifically, variable GMV or WMV in areas of the DLPFC, insular cortex, and IFG were identified as structural markers of individual differences in tendency to forgive in a large sample of healthy young adults. As such, this research demonstrated structural bases for variations in tendency to forgive that is distributed across distinct gray and white matter areas of the brain.

## Materials and Methods

### Participants

Participants were 194 young adults (58 men, 136 women; mean age = 20.29, SD = 1.61, age range: 18–26 years) from Southwest University (SWU), Chongqing, China who volunteered as part of an ongoing project examining associations between brain imaging, creativity and mental health^25,27,59^. All participants were right-handed and physically healthy. None had a history of neurological or psychiatric illness assessed by a self-report questionnaire before scanning. The study was approved by the SWU Brain Imaging Center Institutional Review Board and all methods were carried out in accordance with the relevant guidelines and regulations specified by this committee. In accordance with the Declaration of Helsinki (2008), written informed consent was obtained from all participants.

### Measures

#### Tendency to Forgive Scale (TTF; Brown, 2003)

The TTF is a 4-item scale which assesses individual differences in the tendency to forgive one’s transgression across situations and relationships^7^. Sample items include, “I tend to get over it quickly when someone hurts my feelings.” and “I have a tendency to harbor grudges.”. Participants were instructed to response on a five-point Likert scale that ranges from strongly disagrees (1) to strongly agree (5). The TTF has been demonstrated to have a reasonable internal reliability and high degree of stability over eight weeks in prior study^7^. The Chinese version of TTF was also shown to have good levels of reliability and validity^60,61^. In this study, the TTF had an acceptable internal consistency, α = 0.60.

### MRI Data Acquisition

MR images were obtained on a 3.0-T Siemens Trio MRI scanner (Siemens Medical, Erlangen, Germany). High-resolution T1-weighted anatomical images were acquired using a magnetization-prepared rapid gradient echo (MPRAGE) sequence (TR = 1900ms; TE = 2.25ms; FA = 9°; Slices = 176; Slice thickness = 1.0 mm; Resolution matrix = 256 × 256; Voxel size = 1 × 1 × 1 mm^3^).

### Voxel-Based Morphometry

The MR images were processed using the SPM8 (Wellcome Department of Cognitive Neurology, London, UK) implemented in Matlab 7.8 (MathWorks Inc., Natick, MA, USA). First, each MR image was displayed in SPM8 to screen for artifacts or gross anatomical abnormalities. For better registration, the reorientation of the images was manually set to the anterior commissure. Segmentation of T1 weighted anatomical images into gray matter, white matter and cerebral spinal fluid was completed using the New Segment toolbox in SPM8. Subsequently, we performed Diffeomorphic Anatomical Registration through Exponentiated Lie (DARTEL) algebra in SPM8 for registration, normalization, and modulation^62^. To ensure regional differences in the absolute amount of GM or WM were conserved, the image intensity of each voxel was modulated by Jacobian determinants. Then, registered images were transformed to Montreal Neurological Institute (MNI) space. Finally, the normalized modulated images (GM and WM images) were smoothed with a 10-mm full-width at half-maximum Gaussian kernel to increase signal to noise ratio.

### Statistical analysis

Statistical analyses of GMV and WMV data were performed using SPM8. In the whole-brain analyses, multiple linear regression were used to explore the association between regional GMV and WMV and individual differences in TTF scores. The TFF scores were used as the variable of interest. Global brain GMV, age, and sex were entered as covariates of no interest. To minimize boundary effects around the borders between GM and WM, an absolute threshold masking of 0.2 was used; that is, voxels with gray matter or white matter values lower than 0.2 were excluded from analyses^63^. For all analyses, the cluster-level statistical threshold was set at *P* < 0.05, and corrected at the non-stationary cluster correction^64^ with an underlying voxel level of *p* < 0.001.

### Data Availability

The datasets analyzed during the current study are available from the corresponding author on reasonable request.

## Electronic supplementary material

Supplementary Information
