# Supplementary material for: Brain Structural Bases of Tendency to Forgive: evidence from a young adults sample using voxel-based morphometry
Source: Sci Rep. 2017 Dec 4;7:16856. doi: 10.1038/s41598-017-16868-3 (PMC5715096; doi:10.1038/s41598-017-16868-3)

Supplementary Results to Accompany

Brain Structural Bases of Tendency to Forgive: evidence from a young adults sample using voxel-based morphometry

Haijiang Li[1]*, Qunlin Chen[2,3]*, Jiamei Lu[1], Jiang Qiu[2,3]

[1]Department of Psychology, Shanghai Normal University, Shanghai 200234, China, [2]Key Laboratory of Cognition and Personality (SWU), Ministry of Education, Chongqing 400715, China, [3]Faculty of Psychology, Southwest University, Chongqing 400715, China.

Correspondence authors:

Jiamei Lu, Prof. (lujiameivip@126.com)

Department of Psychology, Shanghai Normal University

No.100 Guilin Rd. Xuhui district, Shanghai, China, 200234

Tel: +86 21 6432 3257

Table S1 Brain regions with significant associations between TTF and cortical thickness.

Brain regions	Side	MNI coordination			NVtxs	Peak T-Value
		x	y	z		
Positive correlations						
**Superior Frontal Gyrus**	R	20	-4	61	203	3.25
IFG	L	-49	10	17	249	3.41
Negative correlation						
Superior Frontal Gyrus	R	19	28	49	134	2.99
IPL	R	45	-49	41	149	2.89
**IFG**	R	44	27	21	166	2.84
**Insular cortex**	R	39	-7	1	121	2.48

Statistical threshold, p < 0.01, uncorrected. TTF, tendency to forgive; IFG, inferior frontal gyrus; Inferior parietal lobule, IPL; NVtxs, number of vertex.

Figure S1 Association between TTF scores and cortical thickness.

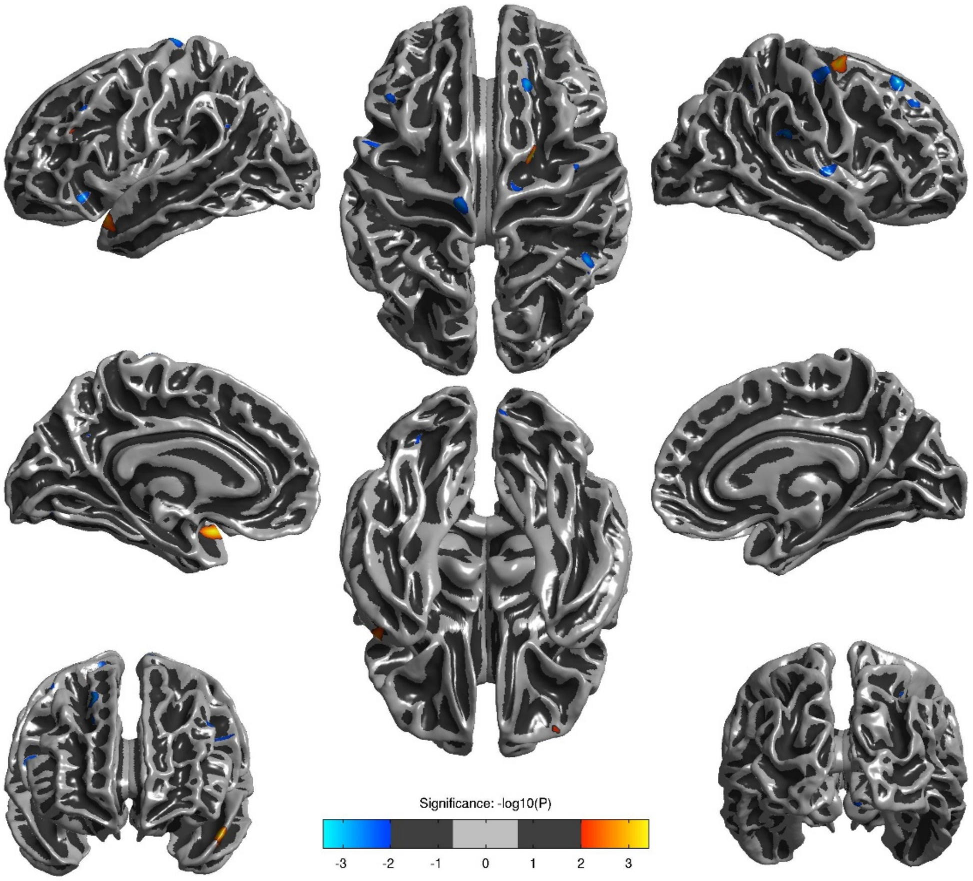

Supplement: Supplementary file 1 — Supplementary Information [file 41598_2017_16868_MOESM1_ESM.pdf]
